# COVID-19: Lockdown and its impact on medical students: A cross sectional study from a medical school in Mauritius

**DOI:** 10.3126/nje.v11i2.36951

**Published:** 2021-06-30

**Authors:** Indrajit Banerjee, Jared Robinson, Poornasha Mohabeer, Abhishek Kashyap, Ananya Shukla, Brijesh Sathian

**Affiliations:** 1-5 Sir Seewoosagur Ramgoolam Medical College, Belle Rive, Mauritius; 6 Department of geriatrics and longterm care, Rumailah Hospital, Doha, Qatar

**Keywords:** SARS-CoV-2, Disease Outbreaks, Pandemics, Psychology, Education, Indian Ocean Islands

## Abstract

**Background:**

The objectives of the study were to identify the psychological impacts of lockdown on medical students due to COVID-19 and to discover the educational perplexities being faced by these students during the lockdown.

**Methods:**

A cross-sectional study was conducted at Sir Seewoosagur Ramgoolam Medical College (SSRMC), Mauritius. Questions were designed after an extensive review of the literature, so as to ensure relevance to meet the objectives of the study.

**Results:**

Out of 700 undergraduate medical students, 663 participated, which equates to a response rate of 95%. 348 (52.5 %) of the students were stationed in their hometown and the remaining 315 (47.5 %) were stationed in Mauritius. 464(70%) of the students suffered from the psychological impacts of lockdown whereas 634(95.6%) of students suffered from the educational impact thereof. Mauritian students suffered a greater educational impact aOR4.236[1.606-11.173]. Psychological impacts aOR 1.280 [0.917-1.789] and educational impacts aOR 2.464 [1.076-5.647] were more prevalent in hometown-based students. Students pursuing their clinical studies had aOR1.219 [ 0.531-2.798] a greater educational impact as compared to preclinical studies.

**Conclusion:**

Lockdown triggered both educational and psychological impacts on medical students. On a psychological basis it was proven that the lockdown induced a feeling of guilt and had a greater psychological impact in pre-clinical students. The COVID-19 situation was simultaneously indicated to be a motivator in the majority of students; however, juxtaposed to this was the fact that various students felt as if they couldn’t study at the same level that they were accustomed to due to the uncertainty of the situation.

## Introduction

The novel coronavirus or (COVID-19) which surfaced in Wuhan, China, in 2019 subsequently led to a pandemic [1]. Health systems are facing collapse and the death tolls are rising [2]. Several nations have declared situations of emergency and have invoked national restrictions of movement under various acts such as the Public Health (Control of Disease) Act 1984 (England) [3]. The above being implemented to abate the exposure and viral transmission resulting in the closure of Universities, lockdown of students in confined quarters and has culminated in exacerbating the complexity of medical studies [4-7]. Practice-based learning is the backbone of education in the training of physicians. Hospitals, clinics, and community services are where future doctors learn aswell as develop their professional identities and develop their orientation to patient-focused care which shapes their practice [8]. The COVID-19 pandemic has had profound impacts on medical education globally. For almost all medical student’s clinical placements stopped as health-care settings focused on the care of patients with COVID-19. Teaching in classrooms and laboratories were also cancelled, leaving students to continue their studies remotely [9].

According to research conducted on epidemics, it has been found that the general population does not adapt well with the abrupt changes brought about by the inception of an infectious disease. Individuals with long term morbidities tend to have an increased deterioration in their mental health as the virtue of falling ill or even dying subsequent to the acquisition of the disease instills great fear as well as anxiety and is sometimes perceived as a death sentence by these individuals. Super imposed to this is the feeling of helplessness and vulnerability to an infectious disease which can cause the mental health of an individual to spiral negatively [4,5]. A recently published meta-analysis shows that the pooled prevalence of anxiety among medical students during COVID pandemic is 28% (95% CI: 22-34%) [10]. Deng J, et al. found that medical student’s severity of anxiety symptoms was associated with decreased willingness to work in the specialties of respiratory medicine and infectious diseases. Psychological problems and professional satisfaction appear to be independent factors that affect medical careers and specialty choices [11]. Harries AJ, et al. reported that nearly 75% of the US medical students in their study agreed that the pandemic had significantly disrupted their medical education. In their study, 84.1% of respondents felt at least some degrees of anxiety. A limitation of this study was the survey response rate (29.5%) [12]. It is therefore imperative that the ramifications on medical education of this unprecedented time are to be understood. It is vital for the circumstances and effect thereof to be studied in this period as it may result in an increased psychological and educational burden on medical students [13].

The objectives of the study were to identify the psychological impact of lockdown on medical students due to COVID-19 and to discover the educational perplexities being faced by these students throughout this period. A substantial dearth of data exists which can quantify the effects and understand the impact of this lockdown on medical students, their studies as well as their mental health. This is the first study on the impact of lockdown on medical students reported from the country Mauritius. It is imperative that this study is used by the global community as it lends an insight into the impact of the lockdown on both an educational and psychological basis of students. The data from this study can be used by institutions of education to gain insight into what their students are experiencing. This study can thus be used to rectify any pitfalls or bolster any areas of weakness to ensure that the losses in education caused by this pandemic can be readily circumvented.

## Methodology

### Study Design and participants

This study is a cross sectional study conducted at Sir Seewoosagur Ramgoolam Medical College (SSRMC), Mauritius between the 30th of May 2020 – 8th June 2020. SSRMC is the first medical college established in Mauritius and is affiliated to the University of Mauritius. Approximately 700 students are pursuing MBBS degrees at SSRMC. A total of 663 students from the first to the tenth semester were enrolled in the study with a response rate of 95%. Cluster sampling was applied to select the participating medical school from the available list of medical schools in Mauritius.

### Data Collection

Google forms was utilized to collect the data and was distributed to all of the current students at SSRMC. Students were invited to partake in the online survey platform via Email, WhatsApp message and Telephone calls. An informed consent was taken from every participant before taking part in the survey.

### Questionnaire Design

Questions were designed after extensive reviews of the literature, so that they were relevant to meet the objectives of the study. The final questionnaire was divided into four sections namely: Demographic details, Psychological aspects, Educational aspects and what after COVID-19. A 5-point Likert Scale consisting of a score from 1-5 according to their agreement to the scale- strongly disagree-1, disagree-2, neutral-3, agree-4 and strongly agree-5 was used to measure the psychological and educational impact of medical students due to COVID-19. In case of negative statements direct coding whereas in case of positive statement reverse coding was used.

### Scoring Scale

There were two domains namely – the psychological and the educational domains which were used to ascertain the impact due to Lockdown. There were eight items used to assess the psychological impact on medical students due to COVID-19. The maximum score in this domain was 40. There were seven statements to assess the educational impact on medical students due to COVID-19. The maximum score in this theme was 35. A total score of 20 and above (Yes) was considered to have a psychological impact on cohorts in the psychological domain and a total score below 20 was considered to have no psychological effects (No). A total score of 18 and above (Yes) in the educational domain was considered to have an educational impact and a total of score of below 18 was considered to have no educational impact (No) on medical students due to lockdown.

### Questionnaire validity

The questionnaire was validated by five subject experts comprised of a panel; consisting of a pharmacologist, a public health expert, a physician, an academician and a medical educationist. The content validity and construct validity were determined by the five experts. The questionnaire was modified based on their comments. The average congruency percentage was 90%.

### Face validity

The questionnaire was initially tested via the use of a pilot study with 10 students, their recommendations were analyzed by all the researchers, after which then the questionnaire was modified. The revised questionnaire was implemented in the primary study [14].

### Reliability analysis

The reliability of the questionnaire was ascertained by Cronbach's alpha. The internal consistency between the items using Cronbach's alpha was found to be 0.717. A Cronbach’s alpha score of 0.721 for the psychological aspect was found after removing 3 questions and a score of 0.685 was noted for educational aspects after removing 2 questions respectively. (Annnex-1)

### Inclusion criteria

All the medical students from each of the College’s ten semesters, approximately 700 of whom are currently pursuing an MBBS degree at Sir Seewoosagur Ramgoolam Medical College, Mauritius were included in the study - out of which 663 participated in this study.

### Exclusion criteria

Any questionnaire which was not complete in all respects was rejected from the study. Students who were not willing to participate or whom had not given their consent to participate in the study were excluded therefrom.

### Outcome Variable

The main outcome variables of the study were the psychological impact and educational impact on medical students due to lockdown.

### Explanatory Variable

The demographics, current location of the students and smart phone usage have been defined at an individual level. Factors which were taken into account on the individual level were Gender (male and female), Nationality (Mauritian, Indian, South African and others), Religion (Hinduism Islam, Christianity, Buddhism, Jainism, Others and Atheism), Level of study (1st Professional, 2nd Professional, 3rd Professional: Final Part I, 4th Professional : Final Part II), Current Location (Away from their hometown, in their hometown), Internet Connectivity (good connectivity, poor connectivity) and daily usage of Smart phone (1-2 hours, 2-4 hours, 4-6 hours, 6-8 hours).

### Ethical clearance

Prior to the study, The Ethical Committee and Institutional Review Board of Sir Seewoosagur Ramgoolam Medical College, Mauritius independently reviewed the research protocols as well as the questionnaire and data collection methods. The Research was subsequently approved to be conducted in accordance to the latest version of, Helsinki - Ethical Principles for Medical Research involving Human Subjects guidelines. Full ethical approval was granted by the Research Ethics Committee (Approval Code- 20-05-03).

### Sample Size Calculation

The prevalence rate of depression was found to be 50.7%, in health care workers due to the lockdown caused by the corona virus (Liu, et al. 2020). Assuming that 51% of the subjects in the population have this depression due to Coronavirus. It would require a sample size of 600 for estimating the expected proportion with a 4% absolute precision and a 95% confidence [15].

### Data management and statistical analysis

The quantitative data was managed and analyzed by use of the Statistical Package for the Social Sciences (SPSS) for Windows Version 24.0. Percentages, 95% Confidence Interval, Chi square test and logistic regression analysis was used to establish the statistical association between variables and to find the adjusted odds ratio. p < 0.05 will be considered as statistically significant.

## Results

A total of 663 undergraduate medical students out of 700 participated in the study which equates to an overall response of 94.71%. The mean age of the students was found to be 21.19 ± 1.881 years SD. Table 1 depicts that 277(58.2%) of the participants were male and 386 (41.8%) were female; 455 (68.6%) were Indian; 133 (20.1%) Mauritian, 68 (10.3%) South African and 7 (1.1%) originated from other foreign countries respectively. The students were from the following professional’s: 1st professional 246 (37.1%), 2nd professional 208 (31.4%), 3rd professional: final part I 107(16.1%) and 4th professional: final part II 102(15.4%). Due to the pandemic 348 (52.5 %) of students were stationed in their hometown and the remaining 315 (47.5 %) were in Mauritius (including Mauritian students). 447 (67.4 %) of the students have good internet connectivity whereas 216 (32.6 %) have poor internet connectivity or are having difficulties with the internet connection. The daily smartphone usage of students shows that 221 (33.3%) of the students spend 4-6 hours on their devices and a smaller cohort of 47 (7.1%) of students spend 1-2 hours on their devices per day. 464(70%) of the students suffered from the psychological impact of lockdown whereas 634(95.6%) of the students suffered from the educational impact of lockdown. (Table 1)

Table 2 depicts Female and male students have equivocally suffered from the psychological impact of this lockdown period. This is evident as 273(70.7%) of the female cohort and 191(69%) of the male cohort agreed that the lockdown had a psychological impact on them respectively. Indian students have suffered from a greater psychological impact throughout this lockdown period as opposed to other nationalities. This is evident as 323 (71%) of Indian students suffered from psychological impact; this figure is proportionally lower in that of Mauritian students where 87(65.4%). South African students exhibit the second highest degree of psychological impact as 48(70.6%). The psychological impact of lockdown was more prevalent in the final part II students as opposed to the other levels of study as 75(73.5%) of the final part II students suffered from it as opposed to the 75(70.1%) of the Final Part I students, 145(69.7%) of the 2nd Professional students and 169(68.7%) of the 1st Professional students whom believed that they suffered psychological impacts from the lockdown.

168(77.8 %) of the students agreed that a poor internet connectivity had a psychological impact, whereas 48(22.2%) of the students who had a poor internet connectivity didn’t suffer from the psychological impact thereof. The psychological impact of poor internet connectivity correlates with that of hours spent on the smart phone device as an increase in the psychological impact is seen with an increase in hours spent on the smart phone. 144(77.8%) of students who spent between 6-8 hours on their smartphone suffered from the psychological impact thereof; whereas a lower 156(70.6 %) of students who spent between 4-6 hours and 137(65.2 %) of students who spent between 2-4 hours on their smartphone suffered from the psychological impact thereof. These findings were found to be statistically significant p<0.05.

Female and male students have almost equivocally suffered from the educational impact of this lockdown period. This is evident as 372(96.4%) of the female cohort and 262(94.6%) of the male cohort agreed that the lockdown had an educational impact on them respectively. Indian Students have suffered from a greater educational Impact throughout this lockdown period as opposed to other nationalities. This is evident as 443 (97.4%) of Indian students agree with this statement; this figure is proportionally lower in that of Mauritian students where 123(92.5%) agree. South African students exhibit the lowest degree of educational impact as a much lower proportion of only 61(89.7%) of the South African students agree that this lockdown had an educational impact. The educational impact of lockdown was more prevalent in the final part I students as opposed to the other levels of study as 103(96.3%) of the final part I students agreed with this premise as opposed to the 98(96.1%) of the Final Part II students, 197(94.7%) of the 2nd Professional students and 236(95.9%) of the 1st Professional students whom believed that they suffered educational impacts from the lockdown. 205(94.9%) of the students agreed that a poor internet connectivity had an educational impact whereas a greater proportion of students 429(96%) of whom had good internet connectivity suffered the educational impact thereof. The educational impact of good internet connectivity correlates with that of hours spent on the smart phone device as a decrease in the educational impact is seen with a decrease in hours spent on the smart phone. 178(96.2 %) of students who spent between 6-8 hours on their smartphone suffered from the psychological impact thereof; whereas a lower 197(93.8%) of students who spent between 2-4 hours on their smartphone suffered from the psychological impact thereof. The lowest educational impact was recorded in 42(89.4 %) of students who spent between 1-2 hours on their phones per day. Students who suffered the most from the educational impact were away from their hometown as 307(97.5%) of students whom were not at home suffered from the educational impact. (Table 3)

Table 4 represents a logistic regression analysis of educational and psychological impacts due to lockdown. It is evident that the Mauritian students suffered from a greater educational impact as opposed to a psychological impact; by virtue of the fact that the educational impact accounted for an adjusted odds ratio of 4.236[1.606-11.173] . Students whom remained in their hometown had a greater educational impact as opposed to the students who remained away from their hometown, it is also evident that the psychological impact aOR 1.280 [0.917-1.789] was more in students whom were located in their hometown as compared to other nationalities but it didn’t reach statistical significance. The educational impact accounted for aOR 2.464 [1.076-5.647]. A good internet connection had greater psychological impacts on students as opposed to educational impacts as the psychological impacts accounted for aOR1.785[1.226-2.600]. Students who used their smartphone devices for 6-8 hours per day had a greater psychological impact as opposed to an educational impact; as the psychological impact accounted for an aOR 2.602[1.326-5.106]. Students pursuing their clinical studies had a greater aOR1.219 [ 0.531-2.798] educational impact as compared to their counterparts whom were in their preclinical years of study but it didn’t reach statistical significance.

**Figure 1.**
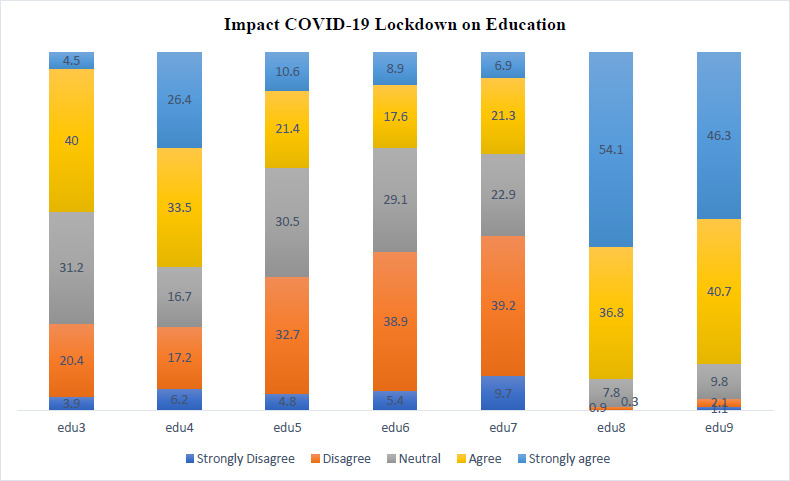
Impact COVID 19 Lockdown on Education

Figure 1 clearly depicts that the majority of students 40% thereof agreed that they did not feel a change in their attitude towards their studies. In the second bar 26.4% of students very strongly agree and a larger cohort of 33.5% agree that online classes are a more efficient method of teaching and learning as opposed to traditional classes. It is evident from the third bar that 32.7% of the students disagree that the current online application being used to conduct online classes is sufficient. The fourth bar clearly depicts that 38.9% of the students disagree to being able to adapt to online teaching, a further 29.1% of students feel neutral towards the adaptation. The 5th bar depicts that 39.2% of students disagree, 9.7% strongly disagree and 22.9% feel neutral towards distraction from online paraphernalia. The 6th bar clearly depicts that the majority 54.1% of students strongly agree and 36.8% of students agree that the lack of exposure to real patients in a real hospital may weaken their clinical skills. The 7th bar depicts similar findings to that of the 6th with the majority 46.3% of students strongly agreeing and 40.7% of students agreeing that decreased exposure to real patients in a real hospital might hinder the future interaction of medical students with their patients.

**Figure 2.**
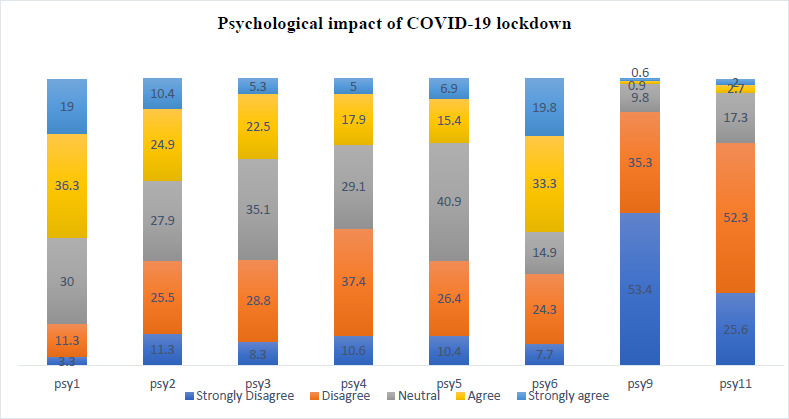
Psychological impact of COVID-19 lockdown

Figure 2 This graph depicts the guilt felt by students due to their level of productivity in the lockdown period, it is evident that a large cohort of 36.3% agreed and 19% strongly agreed to feeling guilty. The 2nd bar clearly depicts that 27.9% of the students felt neutral and 24.9% agreed that they experienced feelings of depression owing to their inability to adapt to this sudden change in their life. 35.1% felt neutral and 28.8% disagree that they managed their time productively to explore some new skills and talents. 37.4% of students disagree and 10.6% of students strongly disagree that lockdown provides them with the opportunity to revise difficult modules and focus on their areas of weakness. 40.9% of students feel neutral, 26.4% disagree and 10.4% of the students strongly disagree that the lockdown has been a pleasant experience. 33.3% of students agree and a further 19.8% strongly agree that they are keeping a normal sleeping pattern throughout lockdown. 53.4% of students strongly disagree and 35.3% disagree that this pandemic has made them proud to have chosen a medical career. 52.3% of students disagree and 25.6% strongly disagree that this pandemic has motivated them to study more diligently so as to be an asset to the medical community.

**Figure 3.**
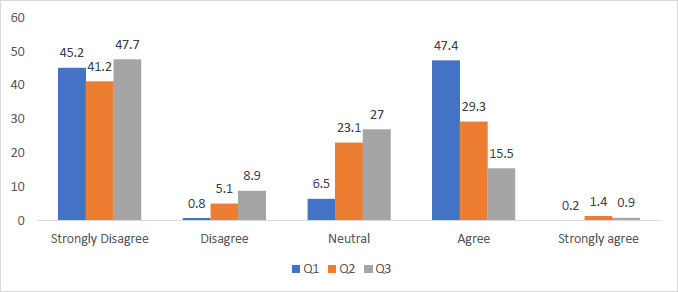
After COVID-19

Figure 3 reveals 45.2% of students strongly disagree whereas 47.4% of students agree that they will make use of safety equipment after the pandemic. 41.2% of students strongly disagree as opposed to the 29.3% of students who agree to maintain a greater distance from their patients in the early post pandemic period. A large cohort of 47.7% of students strongly disagree and a further 8.9% of students disagree; whereas 15.5% of student agree that people might ostracize doctors after treating COVID-19 patients due to the fear of being infected.

**Table:** Annex-1

**Psychological Impact**
Psy1. I have felt guilt due to my level of productivity throughout this lockdown period
Psy2. I have experienced feelings of depression owing to my inability to adapt to this sudden change in my life
Psy3. I am satisfied with my ability to manage my time and use it productively to explore some new skills and talents.
Psy4. Lockdown provides me the opportunity to revise difficult modules and focus on my areas of weakness
Psy5. This lockdown period has been a positive experience
Psy6. I am keeping a normal routine sleeping pattern during this lockdown period.
Psy7. The pandemic has made me more apprehensive with regards to clinical practice**
Psy8. Violence against doctors during this pandemic has made me concerned about my own as well as my family’s safety in the future. **
Psy9 This pandemic has made me proud to have chosen the medical field as a career
Psy10. I am concerned at having to put my life in danger, in case of another pandemic in future**
Psy11. This pandemic has motivated me to study more diligently so as to be an asset to the medical community
** Psy 7, 8, 10 were removed - Reliability analysis: Cronbach’s alpha= 0.721
**Educational impact**
Edu1. I have been unable to study given the amount of stress and panic due to COVID19**
Edu2. This pandemic has ignited my interest in newer fields of medical science, especially those which deal with protection and prevention against these forms of viral disease**
Edu3. I do not feel any change in my attitude towards my studies.
Edu4. The online classes are as efficient compared to traditional classroom method of learning and teaching
Edu5. The current online application that is being used to conduct online classes is sufficient.
Edu6. Students are able to adapt to this new method of teaching
Edu7. I am distracted by other online paraphernalia (games, movies, social media), while classes are being conducted
Edu8 No exposure to real patients in a real hospital may weaken students’ clinical skills. (Negative)
Edu9. Less exposure to real patients in a real hospital might hinder the future interaction of medical students with their patients. (Negative)
** Edu 1, 2 were removed - Reliability analysis: Cronbach’s alpha= 0.685
**What after COVID-19**
Q.1. I will make use of safety equipment like masks and gloves after the pandemic
Q.2. I will maintain a greater distance from my patients in the early post pandemic period
Q.3. I believe people might ostracize doctors after treating COVID19 patients next due to the fear of being infected

## Discussion

Human beings are creatures of habit and prefer to live in a highly organized and structured environment [16]. A source of grave and deep concern as well as unhealthy merited stress [17] is the nuance of untimely and rapid changes in one’s day to day lifestyle. One manner in which we as the human race create a routine and or a structured lifestyle is through the use of habits [18-20]. These habits are used in order to keep the proverbial flow in one’s day to day living constant. Research conducted by Judah G, et al, indicates that this behavior of habit forming is a healthy and intrinsic motivation [21]. The pandemic which ensued because of the coronavirus occurred at an unprecedented rate and has disrupted many of our ingrained and important habitual acts [1]. Several nations have declared states of emergency and have invoked national restrictions of movement [2,3] the mere implication and speed at which these restrictions were invoked completely nullified all standard lifestyle and living normalities. The lockdowns’ being implemented have resulted in the closure of Universities, lockdown of students in confined quarters and has culminated in exacerbating the complexity of medical studies [4-7]. The rapid change and alteration to the daily lives and routines of individuals due to this pandemic is unprecedented. It has caused severe alterations to almost every aspect of each student’s routine and day to day lifestyle.

This research has both shed light as well as simultaneously provided valuable data to a proverbial grey area where a substantial dearth existed prior to this research. The data attained through this research suggests numerous fathomable and corporeal impacts of the lockdown and its effects both in the psychological realm as well as the educational realm of the students. The initial study design pivoted on the basis of the need to simply understand and record the raw impacts of the current lockdown on students on both a psychological and educational basis, however due to the rich yield and nature of the data provided; it has exceeded the initial expectations of the study as intriguing findings such as the impact of this pandemic in the future decisions of students in terms of their choice in specialty has been augmented. The data has also revealed the multifactorial but Intricately, interlinked and “domino effect” that certain aspects of the lockdown exert on the students, thus this study has provided insight far beyond the initial scope of psychology and education. An almost perfect equipoise of data was collected from the students as 348 (52.5%) of the data was sourced from students who were in their hometown and the remaining 315 (47.5%) was obtained from students who were away from their hometown. This spread of input allowed for a richer base of data and, thus allowed for the commission of a greater holistic picture.

A relevant and poignant finding is the increased screen time and usage of smart phones relayed by the students. 30% of the students spend between 6-8 hours per day on their smart phone devices. This is more than one quarter of an entire day which is spent behind a device. Within this cohort of data, it was noted that those students who spent more time on their smart phones had less changes in their attitude towards their studies as compared to students who had a reduced smart phone usage [22]. The increased usage of smart phones also coincided with students who suffered from a higher level of distraction due to online paraphernalia [23]. A correlation was noted between students who experienced a psychological impact due to lockdown and those of whom which had good internet connectivity (52.8%). This trend coincides with the data received from students with regards to the psychological impact due to lockdown as a total of 191(65%) of the male students and 273(70.7%) of the female students felt that they had experienced a psychological impact due to lockdown. This relationship between smartphone use and the lockdowns psychological impact is supported by researched rendered by Aljohara A. Alhassan et al, who identified a definite positive linear relationship between addiction to ones smartphone and depression [24]. Although, the data suggests a correlation between various factors. The findings will be discussed under three subheadings, namely the psychological impact, the educational impact and life after COVID.

### Psychological impact

The lack of productivity and subsequent guilt experienced by students shows a marked resemblance to that of those students suffering from the psychological impact of lockdown owing to their inability to adapt to the sudden change in their life. Female and male students have equivocally suffered from the psychological impact of this lockdown period. This is evident as 273(70.7%) of the female cohort and 191(69%) of the male cohort agreed that the lockdown had a psychological impact on them respectively. This finding is supported by a study conducted in Spain by Ausin B, et al which concluded that the female gender suffered more greatly than their male counterparts due to the lockdown [25].

Research conducted by Yarebeigi H, et al. and team outlines how stress negatively effects learning, their research indicated that the mechanism of how the hippocampus forms long term memories via long term potentiation is altered by the increased cortisol level brought about by stress in foreign situations. This is indicative of what is potentially being experienced by our students throughout this lockdown period [26].

The most resounding finding of this study in the realm of psychology was the fact that the psychological impact of lockdown was more prevalent in the pre-clinical students whom suffered 1.134 times more than their seniors who were in their clinical years of study. This outcome is opposed by findings in research conducted by Satpathy B, et al. which showed the increased psychological impact of the lockdown on final year students in particular [27]. Students in their pre-clinical years of study are generally younger and therefore less mature than their senior counterparts whom are pursuing their clinical years of study. The younger students are therefore greener, less exposed and are less equipped to deal with the challenges and changes faced in difficult circumstances – such as the coronavirus pandemic. This lack of emotional maturity and rigidity is likely the cause for the increased psychological impact faced by the younger students as when compared to their seniors [28-30].

### Educational impact

Medical studies are at the forefront of being one of the most complex and taxing carrier choices. For students to ascertain as well as retain this knowledge the conditions need to fall within a narrow spectrum to optimize their studies. A valid concern in the initial phases of the induction of online lectures from face-to-face and blackboard methods of learning to online lectures was whether students would be able to adapt to such an alteration [31]. It must be noted that 32.3% of Indian students suffer from distractions due to online paraphernalia. This is higher as compared to the 21.8% of Mauritian students and the 14.7% of South African students which are implicated due to this pitfall. The higher ratio of Indian students due to these distractions relates to the proportionally higher rates of the feeling of unproductivity displayed and experienced by Indian students.

The data suggests that this online method of conducting lectures has not been well adopted by the students as 38.9% of the students disagree to being able to adapt to online teaching and a further 29.1% of students feel neutral towards the adaptation. Similar findings were reported by Bacow LS, et al. and Panigrahi R, et al. [32,33] This finding is supported by the research conducted in China by Shuanglan Luo et al, who concluded that the adaptation of students to this online medium was low [34]. Research undertaken by Banerjee I, et al. at Nepal is bolstered and supported by the data as a cohort of the students did not believe online classes were as useful and as rich as the old school in person, black board method of learning. Indian students were found to prize physical old school methods of teaching at a high rate of 86.2%; this is closely followed by the Mauritian cohort at 73.7% and South African cohort at 73.5% [ 35]. This is in direct contrast to the student’s feelings with regards to efficiency as 26.4% of the students very strongly agree and a larger cohort of 33.5% agree that online classes are a more efficient method of teaching and learning as opposed to traditional classes. This is supported by findings in research conducted by Sathish MT, et al, whom concluded that online classes were superior to their traditional counterpart [36].

The overwhelming finding of this study in the realm of education is that students pursuing their clinical years of study suffered from an educational impact 1.219 times greater than their junior counterparts whom are in their pre-clinical years of study. This can be attributed to the fact that much of the clinical studies and teaching occurs in a hospital environment where both tangible patients and equipment are present to allow “didactic learning”. Due to the lockdown students are not only barred from going to hospital, but are rather confined to their homes and therefore learning such physical skills remotely is very challenging, thereby depicting as to why senior students suffer from a great educational impact as opposed to their junior counterparts. Such feelings and impacts faced by students are supported by findings of a study conducted by Liley T, et al. where [CT] Computed tomography scanning competence was taught to radiography graduates in Australia via remote access learning. The students in Australia like our own suffered from a lack of confidence in their skills as well as a decreased satisfaction with remote and online learning. This as coupled with an increased preference of the students for in person live hands on learning [37,38].

The majority 54.1% of students strongly agree and 36.8% of students agree that the lack of exposure to real patients in a real hospital may weaken their clinical skills. Similarly, 46.3% of students strongly agree and 40.7% of students agree that the decreased exposure to real patients in a real hospital setting might hinder the future interactions with their patients. A study by Wilcha RJ, et al. notes the challenges faced due to lockdown yet challenges the above notion and fervently concludes that online teaching is effective and through a holistic approach that students will be well equipped [39]. In complete juxtaposition to the above findings O'Byrne L and his team believe that the changes brought about by this pandemic in the medical curriculum will result in a greater preparedness and hands on approach for students in the future and thus will nullify the concerns of lack of exposure; as the ability of doctors to navigate unforeseen challenges is poignant and thus such a pandemic can be used as a valuable learning vehicle [40]. 52.3% of students disagree and 25.6% strongly disagree that this pandemic has motivated them to study more diligently so as to be an asset to the medical community, thus clearly depicting the negative impact of the lockdown on the education of students.

### Life after COVID

It is evident that life after COVID will induce certain changes, 45.2% of students strongly disagree whereas 47.4% of students agree that they will make use of safety equipment in the post COVID era [41]. The South African cohort of students had a relatively higher strong agreement (58.8%) to the use of safety equipment as opposed to Mauritians (48.1%) and Indians (45.7%) [42]. The impact of the pandemic will extend beyond the simple use of additional personal protective equipment and the implication of health protocols [43]. A large cohort of the students, 467 (70%) will ensure to alter the manner in which they approach their patients in the early post COVID era, [44, 45] this again was very strongly supported by the South African cohort which on average had a margin of 5% greater than the other nationalities. The data indicates that students will attempt to maintain a greater physical distance between themselves and their patients. This finding is contrasted by research conducted by Liu Q and team in China where healthcare workers extended the bounds of their training and safety in order to manage with the influx of patients [46].

Data suggests that 494 (75%) of the students are confident that this pandemic and viruses such as the virulent and deadly nature of COVID-19, will not have a deleterious effect on future students [47] and that this pandemic will not lead to a decrease in the number of students opting for a career in the medical field, despite the possible dangers of such an outbreak occurring in future [48,49]. This trend indicates that there will be changes to one’s everyday life style in the post-covid era, but this will not interfere with patient care and the passion medical students have for their choice of career [50,51].

## Strenghts of the study

The strengths of this study is owed to the fact that the data was collected in a truly trying time for the students, as the initial data was collected whilst Mauritius was undergoing and experiencing the harsh reality of its first wave as well as its first major lockdown, thus providing rich insight into the true state of the students as they were experiencing the lockdown and its effects first hand thereby eliminating recall bias. The fact that the study had a large sample size of 700 students, coupled with the response rate of 95% (663/700) further adds great strength and merit to this study.

## Limitation of the study

The Depression Anxiety Stress Scales-21 (DASS-21) to measure the psychological impact was not used in the study.

## Conclusion

Lockdown triggered both educational and psychological impact on medical students. The overall, holistic impact of the lockdown due to COVID19, can be described to be double edged in nature. It is evident that the lockdown exerted both negative and positive effects in various realms of the student’s lifestyle. On a psychological basis it was proven that the lockdown induced a feeling of guilt and had subsequent psychological impacts in certain students. The realm of education however was far more greatly impacted by the lockdown, the data shed light on the fact that students still regard and value physical, old school teaching methods to a greater degree as opposed to the online teaching medium, however feel that online methods are more efficient. The COVID-19 situation was simultaneously proven to be deleterious to the studies of the majority of students; as proven by the fact that various students felt as if they could not study at the same level that they are accustomed to due to the uncertainty of the situation. The students and staff emulated a great amount of tenacity in forging forward with the online classes on such a rapid scale. In the post COVID-19 era it is noted that students will take active cognizance when it comes to personal protective equipment.

### Future scope of the study

This is a cross sectional study which was conducted on a medical school in Mauritius. A Multicentric study including all the medical schools in Mauritius with higher sample size can be planned in future.

### What is already known on this topic?

A recently published meta-analysis shows that the pooled prevalence of anxiety among medical students during COVID pandemic is 28%. 75% of the US medical students agreed the pandemic had significantly disrupted their medical education but there is a dearth of data from Africa in general and Mauritius in particular.

### What this study adds

This is the first study on the impact of lockdown on medical students with survey response rate of 95% reported from the country Mauritius. It is imperative that this study is used by the global community as it lends an insight into the impact of the lockdown on both an educational and psychological basis of students. The data from this study can be used by institutions of education to gain insight into what their students were experiencing.

## Figures and Tables

**Table 1.** Demographic details

Variables		n, % (95 % CI)
**Gender**	Male	277, 41.8 (38-45.6)
	Female	386, 58.2 (54.4-62)
**Nationality**	Indian	455, 68.6(64.9-72.2)
	Mauritian	133, 20.1 (17.1-23.3)
	Others	7, 1.1(0.4-2.2)
	South African	68, 10.3 (8.1-12.8)
**Religion**	Atheist	13, 2(1.1-3.3)
	Buddhism	1, 0.2(0-0.8)
	Christianity	36, 5.4(3.8-7.4)
	Hinduism	495, 74.7 (71.2-77.9)
	Islam	80,12.1(9.7-14.5)
	Jainism	7, 1.1(0.4-2.1)
	Others	5, 0.8(0.3-1.8)
	Sikhism	26, 3.9(2.6-5.7)
**Level of study**	1st Professional	246, 37.1(33.4-40.9)
	2nd Professional	208, 31.4(27.9-35.1)
	3rd Prof : Final Part I	107, 16.1(13.4-19.2)
	4th Prof : Final Part II	102, 15.1 (12.7-18.4)
**Preclinical/ Clinical studies**	Preclinical	454, 68.5 (64.8-72 )
	Clinical	209, 31.5 (28-35.2 )
**Current Location**	Away from the hometown	315, 47.5(43.7-51.4)
	Hometown	348, 52.5(48.6-56.4)
**Internet Connectivity**	Good connectivity	447, 67.4 (63.7-77)
	Poor connectivity	216, 32.6(29-36.3)
**Daily usage of Smart phone**	1-2 hours	47, 7.1(5.3-9.3)
	2-4 hours	210, 31.7(282-35.4)
	4-6 hours	221, 33.3(29.8-37.1)
	6-8 hours	185, 27.9(24.5-31.5)
**Psychological impact**	Yes	464, 70(66.3-73.5)
	No	199, 30(26.6-33.7)
**Educational impact**	Yes	634, 95.6(93.8-97.1)
	No	29, 4.4(3-6.2)

**Table 2.** Association between psychological impact on medical students due to lockdown and demographic details

		Psychological Impact		P value
		Yes	No	
**Gender**	Male	191(69)	86(31)	0.688^x^
	Female	273(70.7)	113 (29.3)	
**Nationality**	Indian	323(71)	132(29)	
	Mauritian	87(65.4)	46(34.6)	0.498^X^
	Others	6(85.7)	1(14.3)	
	South African	48(70.6)	20(29.4)	
**Religion**	Atheist	9 (69.2)	4(30.8)	
	Buddhism	1(100)	0(0)	
	Christianity	27(75)	9(25)	0.768^x^
	Hinduism	341(68.9)	154(31.3)	
	Islam	55(68.8)	25(31.3)	
	Jainism	5(71.4)	2(28.6)	
	Others	4(80)	1(20)	
	Sikhism	22(84.6)	4(15.4)	
**Level of study**	1st Professional	169(68.7 )	77(31.3 )	
	2nd Professional	145 (69.7)	63 (30.3 )	0.847^x^
	3rd Prof : Final Part I	75(70.1)	32(29.9)	
	4th Prof : Final Part II	75(73.5)	27(26.5)	
**Preclinical/clinical studies**	Preclinical	314(69.2)	140(30.8)	0.524X
	Clinical	150(71.8)	59(28.2)	
**Current Location**	Away from the hometown	229(72.7)	86(27.3)	0.150^x^
	Hometown	235(67.5 )	113(32.5 )	
**Internet Connectivity**	Good connectivity	296(66.2 )	151(33.8 )	0.002**
	Poor connectivity	168(77.8 )	48(22.2 )	
**Daily usage of Smart phone**	1-2 hours	27(57.4 )	20(42.6 )	
	2-4 hours	137(65.2 )	73 (34.8 )	0.01**
	4-6 hours	156(70.6 )	65(29.4 )	
	6-8 hours	144(77.8 )	41 (22.2 )	

P<0.05**- statistically significant

**P>0.05^X^- statistically insignificant**

**Table 3.** Association between educational impact on medical students due to lockdown and demographic details

		Educational Impact		P value
		Yes	No	
**Gender**	Male	262(94.6)	15(5.4)	0.336^x^
	Female	372(96.4)	14 (3.6)	
**Nationality**	Indian	443 (97.4)	12(2.6)	
	Mauritian	123(92.5)	10(7.5)	0.006**
	Others	7(100)	0(0)	
	South African	61(89.7)	7(10.3)	
**Religion**	Atheist	11 (84.6)	2(15.4)	
	Buddhism	1(100)	0(0)	
	Christianity	33(91.7)	3(8.3)	0.001**
	Hinduism	479(96.8)	16(3.2)	
	Islam	74(92.5)	6(7.5)	
	Jainism	7(100)	0(0)	
	Others	3(60)	2(40)	
	Sikhism	26(100)	0(0)	
**Level of study**	1st Professional	236(95.9)	10(4.1 )	
	2nd Professional	197(94.7)	11(5.3)	0.891^x^
	3rd Prof : Final Part I	103(96.3)	4(3.7)	
	4th Prof : Final Part II	98(96.1)	4(3.9)	
**Preclinical/clinical studies**	Preclinical	433(95.4)	21(4.6)	0.690^x^
	Clinical	201(96.2)	8(3.8)	
**Current Location**	Away from the hometown	307(97.5)	8(2.5)	0.028**
	Hometown	327(94)	21(6)	
**Internet Connectivity**	Good connectivity	429(96)	18(4)	0.002**
	Poor connectivity	205(94.9)	11(5.1)	
**Daily usage of Smart phone**	1-2 hours	42(89.4 )	5(10.6)	
	2-4 hours	197(93.8 )	13 (6.2 )	0.021**
	4-6 hours	217(98.2 )	4(1.8 )	
	6-8 hours	178(96.2 )	7 (3.8 )	

P<0.05**- statistically significant

**P>0.05^X^- statistically insignificant**

**Table 4.** Logistic Regression analysis of Educational and Psychological impact due to Lockdown

		Psychological Impact Adjusted Odds Ratio (aOR) and 95% Confidence Interval	Educational Impact Adjusted Odds Ratio (aOR) and 95% Confidence Interval
**Gender**	Female	1	1
	Male	1.088(0.777-1.522)^x^	1.521(0.722-3.205)^X^
**Nationality**	Indian	1	1
	Mauritian	1.020(0.523-1.784)^X^	4.236(1.606-11.173)**
	Others	0.788(0.419-1.483)^x^	1.411(.512-3.889)^X^
	South African	2.5(0.283-22.123)^X^	-
**Religion**	Atheist	1	1
	Buddhism	0.409 (0.84-2.002)^X^	-
	Christianity	-	1
	Hinduism	0.545(0.148-2.012)^x^	-
	Islam	0.403(0.136-1.188)^X^	-
	Jainism	0.400(0.125-1.283)^x^	-
	Others	0.455(0.064-3.212)^x^	1
	Sikhism	0.725(0.64-8.315)^x^	-
**Level of study**	1st Professional	1	1
	2nd Professional	0.790(0.472-1.324)^x^	0.963(.295-3.145)^X^
	3rd Prof : Final Part I	0.829(0.488-1.408)^x^	0.731(.227-2.355)^X^
	4th Prof : Final Part II	0.844(0.461-1.544)^X^	1.051 (0.256-4.319)^X^
**Clinical/Preclinical**	Preclinical	1.134(0.790-1.623)^X^	1
	Clinical	1	1.219 (0.531-2.798)^X^
**Current Location**	Away from the hometown	1	1
	Hometown	1.280(0.917-1.789)^X^	2.464(1.076-5.647)**
**Internet Connectivity**	Good connectivity	1.785(1.226-2.600)**	1
	Poor connectivity	1	1.279(0.593-2.757)^X^
**Daily usage of Smart phone**	1-2 hours	1	1
	2-4 hours	1.390(0.730-2.648)^X^	0.33(.1-1.092)^X^
	4-6 hours	1.778(0.931-3.394)^X^	0.596(.233-1.527)^X^
	6-8 hours	2.602(1.326-5.106)**	2.133(0.615-7.405)^X^

P<0.05**- statistically significant

**P>0.05^X^- statistically insignificant**
